# Data on chief financial officer attributes and risk management strategies for Nigerian listed financial institutions

**DOI:** 10.1016/j.dib.2019.104609

**Published:** 2019-10-04

**Authors:** Alex Adegboye, Stephen A. Ojeka, Kofo Adegboye, Oluwaseyi Alabi, Mosinmileoluwa Afolabi, Francis Iyoha

**Affiliations:** a Department of Accounting, Covenant University, Ota, Ogun State, Nigeria; b Department of Accounting, Obafemi Awolowo University, Ile-Ife, Osun State, Nigeria; c Department of Finance, Covenant University, Ota, Ogun State, Nigeria

**Keywords:** Data, CFO characteristics, Enterprise risk management, Firm characteristics

## Abstract

This data article is related to the research article “Chief Financial Officer Roles and Enterprise Risk Management: An Empirical Based Study” Ojeka et al. (2019). The study explores the impact of CFO roles on the execution of ERM strategies sampled with Nigerian financial institutions for the period of 2013–2017. The study develops three distinct indexes representing the CFO roles namely CFO power, CFO experience and CFO knowledge via principal component factoring. The study also measures ERM components simultaneously to capture the extent of sophisticated ERM system. This article presents the data collected from 33 financial companies listed on Nigerian stock exchange. The data were extracted from the annual reports of the sample companies using quantitative content analysis about Enterprise Risk Management, Chief Finance Officer Roles and Firm level data.

**Table:** Specifications Table

Subject area	*Accounting, Finance*
More specific subject area	*Corporate Governance*
Type of data	*Excel file*
How data was acquired	*Extracted from the annual report of respective Nigerian listed financial institutions*
Data format	*Raw data, Filtered*
Experimental factors	*The firm sample is based on the financial institutions listed on the Nigerian stock exchange*
Experimental features	*Panel data of two measures – Chief financial officer attributes and Risk management strategies for the period*
Data source location	*Nigeria*
Data accessibility	*The data are attached to this article.*
Related research article	*A relevant research article is* Ojeka, S. A. et al. (2019) ‘Chief financial officer roles and enterprise risk management: An empirical based study’, *Heliyon*. Elsevier Ltd, 5(6), p. e01934. https://doi.org/10.1016/j.heliyon.2019.e01934[1].

**Table:** 

**Value of the Data** • The dataset provides firm characteristics indicators used in the sample for the period 2013–2017. The dataset encompasses CFO attribute score calculated using Principal Component Analysis, enterprise risk management and firm financial data. • The data can be used to evaluate the dimensions of enterprise risk management in Nigerian context at the firm level. The research could unveil the sensitivity of risk management strategies to firm level and country level characteristics. • The dataset is useful for researchers to explore the different corporate governance dynamics especially from the CFO perspective and firm's performance. Future studies can explore the bi-dimensional relationship between corporate governance, CFO attributes, firm performance and enterprise risk management.

## 1 Data

The dataset consists of 165 year-firm observation extracted from the thirty-three (33) financial institutions listed on Nigerian Stock Exchange during 2013–2017. We consider five (5) years from 2013 to 2017 because the period covers the fully implementation and disclosures of financial institutions Risk Management following the Central Bank of Nigeria directives. Table 1 shows the descriptive data on enterprise risk management while the subsequent Table 2 reveals the proxy for CFO attributes and Table 3 depicts the data relating to firm performance and other firm-level characteristics.

**Table 1:** Risk management data descriptive.

Variables	Data type	Description
*RiskReport*	Binary (Yes/No)	Dataset shows 1 if the annual report of a firm includes risk management report and 0 otherwise
*CROx*	Binary (Yes/No)	Dataset takes 1 if the firm designated risk management to specific committee and 0 otherwise
*RCommittee*	Binary (Yes/No)	Dataset takes 1 if the firm designated risk management to specific committee and 0 otherwise
*RBoard*	Binary (Yes/No)	Dataset represents 1 if the sole responsibility of risk management is saddled with the corporate governance that is the board of director and 0 otherwise.
*Rfrequency*	Binary (Yes/No)	Dataset shows 1 if the firm at least biannually performs risk assessment and 0 otherwise.
*Rlevel*	Binary (Yes/No)	Dataset is 1 if the company carries out the risk assessment procedure at a level lower than the overall company and 0 otherwise
*Rmethod*	Binary (Yes/No)	Dataset takes 1 if the company adopts both qualitative and quantitative methods of risk assessment, and 0 otherwise
*ERMsco*	Continuous	The aggregate of the following RiskReport, CROx, RiskCommittee, RCtoBoD, RAfrequency, RAlevel, RAmethod.
*ERMsopH*	Binary (Yes/No)	Dummy variable equal to 1 if ERMsco is equal to or higher than 4, and 0 otherwise using for Logistics Regression

**Table 2:** CFO attributes data descriptive.

Variables	Data type	Description
*Expertise*	Binary (Yes/No)	Dataset equals 1 if the CFO possesses Recognized Accounting Professional Qualification, 0 otherwise
*AuditExp*	Binary (Yes/No)	Data equals 1 if the CFO has some experience in auditing during his/her career, 0 otherwise
*Education*	Binary (Yes/No)	Data equals 1 if the CFO has Master of Business Administration, and 0 otherwise
*ConsultExp*	Binary (Yes/No)	Data equals 1 if the CFO has some experience in consultancy during his/her career, 0 otherwise
*Directorship*	Binary (Yes/No)	Data equals 1 if the CFO is on the Board, 0 otherwise
*CFO gender*	Binary (Yes/No)	Data equals 1 if the CFO is male, 0 otherwise
*Retention*	Binary (Yes/No)	Data equals 1 if the CFO is the same person in YEAR_t_ as in YEAR_(t-1)_, 0 otherwise

**Table 3:** Firm level data descriptive.

Variables	Data type	Description
*ROA*	Ratio	Profit after Taxation divided by Total Asset of the firm
*Tobin Q*	Ratio	The book value of total assets minus the book values of equity plus the market value of equity all divided by the book value of total assets.
*Leverage*	Ratio	Total Debt/Total Common Equity
*Firm size*	Numeric	The natural logarithm total assets of the firm
*Board Magnitude*	Count	The total number of members on the board

## 2 Experimental design, materials, and methods

We obtained ERM measure using a two-step approach in line with Florio & Leoni (2016) [2]. The first approach aggregates all the identified ERM indicators components, which include if the annual report of a firm includes risk management report (*RiskReport)*, if firms have CRO *(CROx)*, if the firm designated risk management to specific committee (*RCommittee),* if the sole responsibility of risk management is saddled with the corporate governance that is the board of director *(RBoard)*, if the firm at least biannually performs risk assessment (*Rfrequency),* inclusive risk assessment procedure *(Rlevel),* and if the firm adopts both qualitative and quantitative methods (*Rmethod).* The ERM score (*ERMsco*) ranges from 0 to 6, then we adopt a binary variable for *ERMsopH*, which equals 1 if *ERMsco* is higher than 3 and 0 otherwise.

As mentioned earlier, this study emphasizes on the CFO attributes as the basic explanatory variable of ERM. We adopt quantitative content analysis to obtain the CFO characteristics from annual reports. Utilizing data available publicly, we gather data relating to the CFO attributes such as CFO educational background that if the CFO possesses MBA or equivalent, the CFO professional experience (i.e. has experience in auditing or consultancy) with possession of any accounting professional certification (ACA, ACCA or CPA). These data tend to reveal the CFO with or without managerial competence (and with or without accounting background. We also examine the level of CFO directorship that is if the CFO is on the board or not. In addition, one variable related to the CFO gender, another variable on CFO directorship and CFO retention. We obtain the data for the firm's characteristic like the firm size (*Size);* firm leverage (*leverage; TobinQ; and* the return on asset (*ROA)*. We identify data for Board size.
